# Determination of Important Topographic Factors for Landslide Mapping Analysis Using MLP Network

**DOI:** 10.1155/2013/415023

**Published:** 2013-12-18

**Authors:** Mutasem Sh. Alkhasawneh, Umi Kalthum Ngah, Lea Tien Tay, Nor Ashidi Mat Isa, Mohammad Subhi Al-batah

**Affiliations:** ^1^Imaging and Computational Intelligence (ICI) Group, School of Electrical & Electronic Engineering, Universiti Sains Malaysia, Engineering Campus, 14300 Nibong Tebal, Penang, Malaysia; ^2^Department of Computer Science, Faculty of Science and Information Technology, Jadara University, Irbid 21110, Jordan

## Abstract

Landslide is one of the natural disasters that occur in Malaysia. Topographic factors such as elevation, slope angle, slope aspect, general curvature, plan curvature, and profile curvature are considered as the main causes of landslides. In order to determine the dominant topographic factors in landslide mapping analysis, a study was conducted and presented in this paper. There are three main stages involved in this study. The first stage is the extraction of extra topographic factors. Previous landslide studies had identified mainly six topographic factors. Seven new additional factors have been proposed in this study. They are longitude curvature, tangential curvature, cross section curvature, surface area, diagonal line length, surface roughness, and rugosity. The second stage is the specification of the weight of each factor using two methods. The methods are multilayer perceptron (MLP) network classification accuracy and Zhou's algorithm. At the third stage, the factors with higher weights were used to improve the MLP performance. Out of the thirteen factors, eight factors were considered as important factors, which are surface area, longitude curvature, diagonal length, slope angle, elevation, slope aspect, rugosity, and profile curvature. The classification accuracy of multilayer perceptron neural network has increased by 3% after the elimination of five less important factors.

## 1. Introduction 

Landslide is one of the most aggressive natural disasters that causes loss of lives and billions of dollars worth of damages annually worldwide [1]. Landslide is also a frequent problem throughout most of Malaysia following a heavy rainfall. The total economic loss due to landslides in Malaysia reported from 1973 until 2007 is estimated to be about one billion US dollars [2]. Considerable amount of research works have been conducted over the past years to identify the most important factors that cause the slope instability [3]. However, there are different factors such as geological, topographical, and human causes (disregard for sustainable developments) contribute towards landslide occurrences [4, 5].

**Figure 1:**
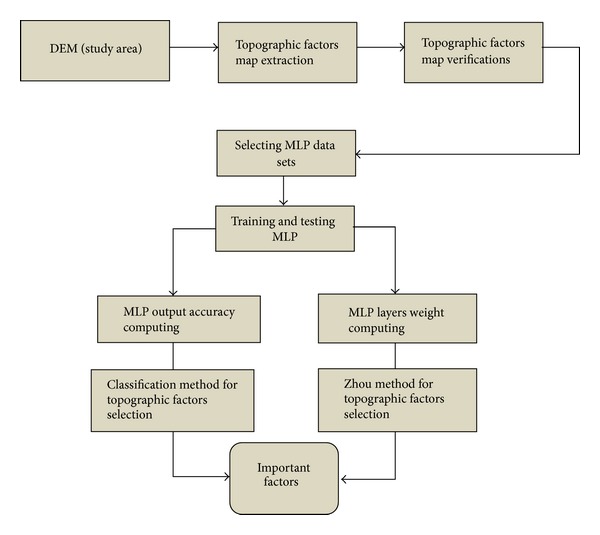
The flow chart of the work methodology.

A literature review of landslide-causing factors shows that topographic factors are linked strongly with landslide occurrence [6–14]. Slope angle, slope aspect, plan curvature, profile curvature and general curvature, are the conventional topographic factors which are extracted from digital elevation model [15]. DEM has recently found widespread application in geographic information system [16] and landslide hazard mapping. Some studies have merged the DEM to landslide hazard mapping in their applications [3, 6, 7, 17] Neural networks have gained popularity from their simplicity, generality and easy application. They have shown good performance when used in landslides prediction and weight determination of the landslide causative factors [18, 19]. One of the most popular neural networks is the multilayer perceptron network. Many training and learning algorithms have been found to improve the performance of the MLP; the most popular one is the back-propagation algorithm. In the year 1999, Zhou has introduced an algorithm to determine the weights of each of the input factors through the neural network training. The study in this paper has many contributions. Firstly, digital elevation model with very high resolution of 5 meters/pixel is used, while the previous studies used 20 to 10 meters/pixel resolution. Secondly, this study includes the extraction of new topographic factors, which has not been performed on Penang island or in Malaysia before. These seven new factors are cross curvature, tangent curvature, longitude curvature, surface area, surface roughness, rugosity, and diagonal length. Thirdly, the importance of factors is determined using the MLP network layer weights (Zhou method) and output accuracy. Dominant factors which have higher influences towards landslide are determined based on these two methods, that is, weights computed using Zhou method and output accuracy. The dominant factors are used in the landslide hazard analysis for better accuracy.  Figure 1 shows the work methodology for this study.

## 2. Study Area 

**Figure 2:**
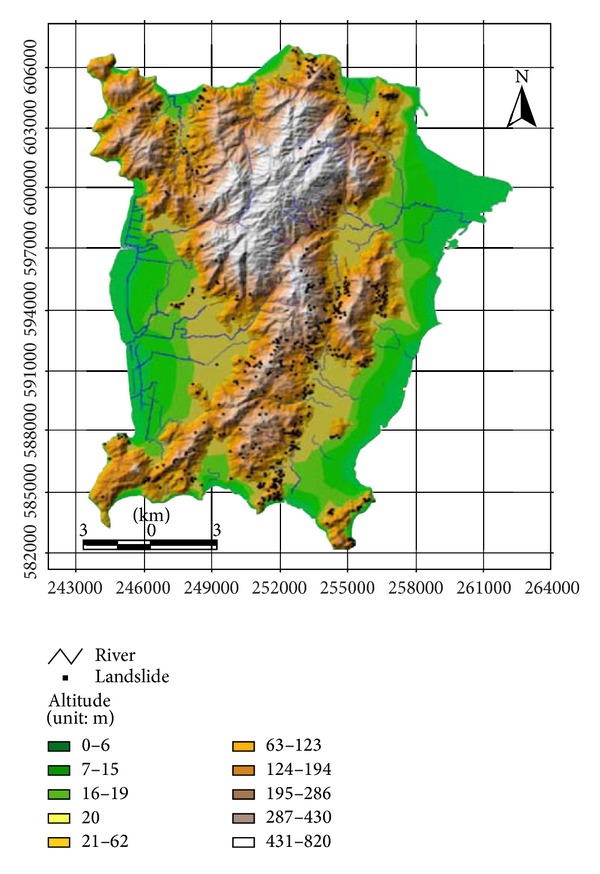
Study area map and landslide location map with hill shaded map.

Penang consists of the island of Penang and a coastal strip on the mainland known as the Province Wellesley.  Figure 2 shows the study area of Penang island and landslide location map with hill shaded map [20]. It lies between 5° 15′ to 5° 30′ N latitude and 100° 10′ to 100° 20′ E longitude. The North Channel separates the study area from the mainland. Penang island occupies an area of 285 km² and it is one of the 13 states of Malaysia, located in the northwest of the Malaysian Peninsula. Topographic elevations vary between 0 m and 820 m above sea level. The geological data of study area shows that Ferringhi granite, Batu Maung granite, clay, and sand granite represent more than 72% of the study area's geology. The rainfall plays a major role in triggering the landslides in the study area. The rainfall amount varies approximately between 2254 mm and 2903 mm annually in the study area. The slope angle ranges from 0° to 87° while 43.28% of Penang island is flat. This research work focuses only on the island, where frequent landslides have occurred and threaten lives and damage properties. Landslides analyses in Penang island have been analyzed by different methods such as statics, fuzzy, and neural network methods [21]. The previous studies used the geological factors and topographic factors, together with other factors, to produce the landslide hazard map. For this research work, the topographic factors are the subject of the study.

## 3. MLP with Back-Propagation Algorithm

**Figure 3:**
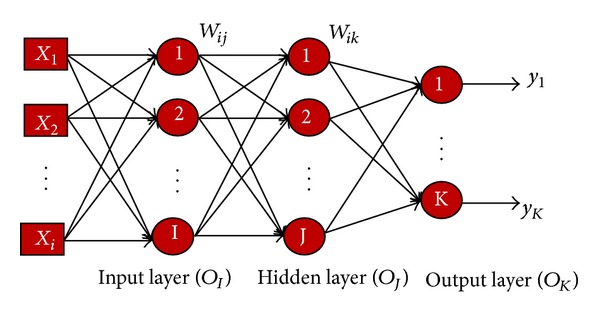
The conventional MLP networks.

The multilayered perceptron is one of the widely used tools in solving classification and prediction problems. This is because of its computational simplicity, finite parameterization, stability, and smaller structure size for a particular problem compared with other neural network structures [22]. MLP consists of a set of layers, namely, input layer, one or more hidden layers, and an output layer (Figure 3).

Each layer in the MLP consists of independent processing units called neurons. These neurons are linked to neurons in other layers through the weight. The network determines the relationship between pairs of input (factors) and output (responses) vectors by altering the weight and bias values. Adjusting the weights between the neurons without a learning algorithm is a difficult task. For that, the back-propagation learning algorithm with momentum was used in this study to reduce the error rate between the actual output and the neural network output results. The algorithm was also used to build up the weight for the input factors [23]. In the input layer, each input is multiplied by a corresponding initial weight; the sum of the product is obtained and then processed by using an activation function to produce a result. For one hidden layer of the MLP network, as shown in Figure 3, the input and output of the  *j*th neuron in the hidden layer are given by (1) and (2), respectively. Consider

(1) $$\begin{array}{c} {\text{net}}_{j}=\sum_{i=1}^{I}w_{ij}\cdot o_{i}, \end{array}$$

where net_*j*_ indicates a hidden layer input, *i* and *j* are indices of different neurons in the network, *I* is the size of the input vector, *w* is the weight, and *O* is the input element. Each neuron of the hidden layer takes its input net_*j*_ and uses it as the argument for a function and produces an output *O*_*j*_ given by:

(2) $$\begin{array}{c} O_{j}=f\left({\text{net}}_{j}\right). \end{array}$$

The function *f* is usually a nonlinear sigmoid function that is applied to the weighted sum of inputs before the signal propagates to the next layer. One advantage of a sigmoid function is that its derivative can be expressed in terms of the function itself, as shown in the following equation:

(3) $$\begin{array}{c} \tilde{f\;}\left({\text{net}}_{j}\right)=f\left(\text{net}\right)\left(1-f\left({\text{net}}_{j}\right)\right). \end{array}$$

An error of training input pattern can be defined as being the difference between the network output, *O*_*k*_, and the target output value, *d*_*k*_, as follows:

(4) $$\begin{array}{c} e_{k}=\left(d_{k}-o_{k}\right). \end{array}$$

The sum of squared error can be calculated as follows:

(5) $$\begin{array}{c} E=\frac{1}{2}\;\sum_{k=1}^{K}e_{k}^{2}, \end{array}$$

where *K* is the number of neurons in the output layer. The error is propagated back through the neural network and is minimized by adjusting the weights between layers. The weight adjustment is expressed as follows:

(6) $$\begin{array}{c} {\Delta w}_{ij}\left(n+1\right)=\eta \left(\delta_{j}\cdot o_{i}\right)+\alpha {\;\Delta w}_{ij}\left(n\right), \end{array}$$

where Δ*w*_*ij*_(*n* + 1) and Δ*w*_*ij*_(*n*) are weight changes in epochs (*n* + 1) and (*n*), respectively, *η* is the learning rate parameter, *δ* is an index of the rate of change in the error, and *α* is the momentum coefficient. This process of feeding forward signals and returns is repeated iteratively until the error of the network is minimized as a whole or reaches an acceptable value, which is 0.1 for this study.

## 4. Methodology

In this research paper, twelve topographic factors relevant to landslide analysis were extracted from the DEM using Matlab software. These factors were analysed for the importance rating of factors by using two different methods, that is, MLP network layers weights (Zhou method) and output classification accuracy. Finally, the important factors selected based on the two different methods were analysed and compared. The DEM map of the study area represents the elevation of Penang island (Figure 5(a)). The DEM was used to extract the maps of twelve topographic factors, which are slope angle, slope aspect, general curvature, plan curvature, profile curvature, cross curvature, tangent curvature, longitude curvature, surface area, surface roughness, rugosity, and diagonal length.

**Figure 4:**
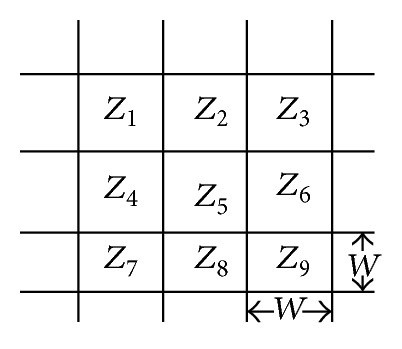
3 × 3  moving window.

Landslide locations of the study area were collected from various government agencies.  Figure 4 shows the moving window and *W* denotes the grid resolution, which is equal to 5 meters in this study. Let  *Z* = *f*(*x*, *y*)  be a given point in DEM surface while *Z*_*i*_(1 ≤ *i* ≤ 9) denotes the elevation at each cell of the 3 × 3 moving window. The extraction algorithms were developed for the twelve topographic factors based on the equations listed in the following sections.

### 4.1. Topographic Factors Extraction

**Figure 5:**
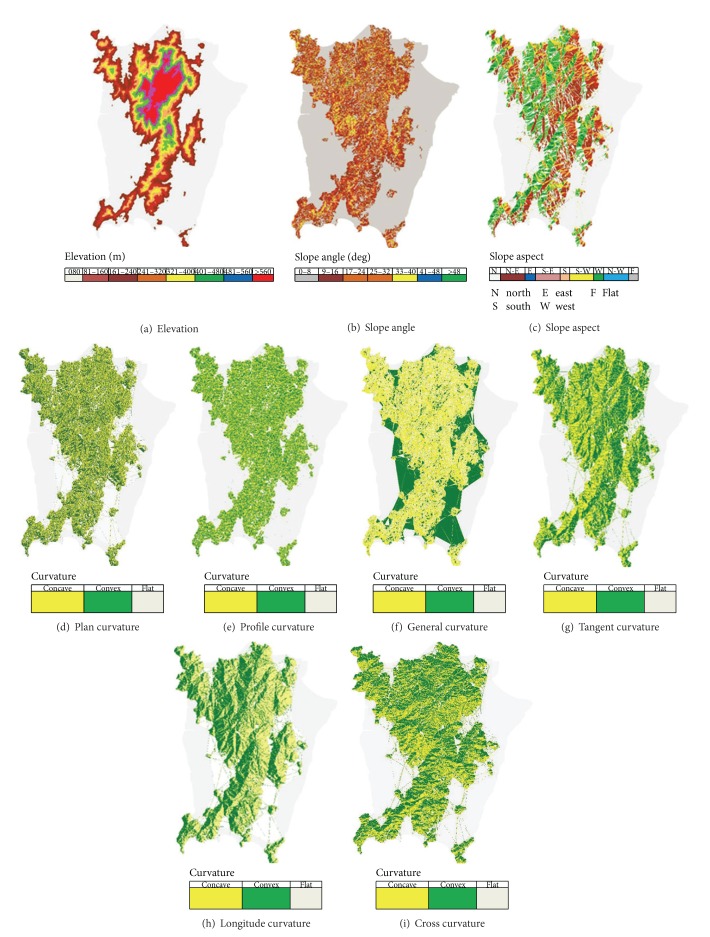

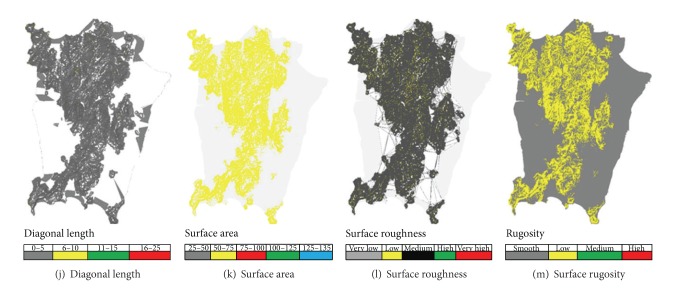
Maps of extracted topographic factors.

*Slope Angle*. The Simple Difference method [24] was applied to extract the slope angles of Penang island.  Figure 5(b) shows the extracted slope angle map with the deviation of the angle level using the following equations:

(7) $$\begin{array}{c} f_{x}=\frac{Z_{8}-Z_{2}}{2W},\;\;f_{y}=\frac{Z_{6}-Z_{4}}{2W}, \end{array}$$

(8) $$\begin{array}{c} \text{slope}\;\text{angle}=\arctan\sqrt{f_{x}^{2}+f_{y}^{2}}. \end{array}$$

*Slope Aspect*. Slope aspect is defined as the direction of the slope. Results from previous research have shown that there is a link between the slope aspect and its prone towards landslide. Furthermore, in some landslide cases, researchers have agreed that the slope aspect is one of the main reasons for the occurrences of landslides [25, 26]. In this study, the slope aspects of Penang island were extracted from the DEM by applying (9) [27]. The slope aspect has been divided into nine classes (Figure 5(c)), namely, North, North East, East, South East, South, South West, West, North West, and Flat:

(9) $$\begin{array}{c} \text{aspect}=270^{{}^{\circ}}+\arctan\left(\frac{f_{y}}{f_{x}}\right)-90^{{}^{\circ}}\frac{f_{x}}{\left|f_{y}\right|}. \end{array}$$

*Curvature*. Surface curvature at a point is the curvature of a line formed by the intersection of the surface with a plane with a specific orientation passing through this point [28–30]. Plan curvature, profile curvature, tangential curvature, longitudinal curvature, cross section curvature, and general curvature are the six types of curvatures [31] which are considered in this paper.

The value of the curvature can be either above, below, or equal to zero, representing the convex, concave, or flat shaped curvatures, respectively, as seen in (15). Some equations and definitions are identified in (10)–(15) before the extraction process. Curvature maps are shown in Figures 5(d)–5(j). More details about the derivation of, *r*, *p*, *t*, and *s* are found in [28]:

(10) $$\begin{array}{c} q=\frac{Z_{1}+Z_{2}+Z_{3}-Z_{7}-Z_{8}-\;Z_{9}}{6W}, \end{array}$$

(11) $$\begin{array}{c} r=\frac{\left(Z_{1\;}+Z_{3\;}+Z_{4\;}+Z_{6}+Z_{7}+Z_{9}-2\left(Z_{2}+Z_{5}+Z_{8}\right)\right)}{3W^{2}}, \end{array}$$

(12) $$\begin{array}{c} p=\frac{\left(Z_{3\;}+Z_{6\;}+Z_{9}-Z_{1}-Z_{4}-\;Z_{7}\right)}{6W}, \end{array}$$

(13) $$\begin{array}{c} t=\frac{\left(Z_{1\;}+Z_{2\;}+Z_{3\;}+Z_{7}+Z_{8}+Z_{9}-2\left(Z_{4}+Z_{5}+Z_{6}\right)\right)}{3W^{2}}, \end{array}$$

(14) $$\begin{array}{c} s=\frac{\left(Z_{3}+Z_{7}-Z_{1}-\;Z_{9}\right)}{4W^{2}}, \end{array}$$

(15) $$\begin{array}{c} \text{curvature}\;\text{value}=\left\{\begin{array}{cc} \text{convex}\;\text{value} & >0\\ \text{concave}\;\text{value} & <0\\ \text{Flat} & \text{elsewhere} \end{array}\right\}, \end{array}$$

where *S* = slope  angle, $\overset{-}{s}=\text{mean}\;\text{slope}$ in 3 × 3 moving window, and *W* = cell  size.

*Plan Curvature*. Plan curvature is defined as curvature in a horizontal plane. In addition, a plan curvature can be defined as the hypothetical line, which crosses a specific cell on the contour line. Plan curvature is derived using the following equation:

(16) $$\begin{array}{c} \text{plan}\;\text{curvature}=\frac{\left(\left(Z_{4}+Z_{6\;}\right)/2-Z_{5}\right)\;}{2w}. \end{array}$$

*Profile Curvature*. Profile curvature is the curvature of the surface in the direction of the steepest slope (with respect to the vertical plane of a flow line). The profile curvature affects the flow velocity of water draining the surface and influences erosion and deposition. In locations with convex (negative) profile curvature, the erosion will prevail while depositions occur in locations with concave (positive) curvature [31]. The following eqaution was used to calculate the profile curvature for this study:

(17) $$\begin{array}{c} \text{profile}\;\text{curvature}=\frac{\left(\left(Z_{2}+Z_{8\;}\right)/2-Z_{5}\right)\;}{2w}. \end{array}$$

*General Curvature*. As identified by Wood [32, 33], general curvature (also called total curvature) is the curvature of the surface itself (not the curvature of a line formed by the intersection of the surface with a plane). The general curvature can be positive or convex (indicating peaks), negative or concave (indicating valleys), or zero (indicating flat surface or a saddle) [31]. Taking into consideration the previous kinds of curvatures, a link can be established with general curvature as follows:

(18) $$\begin{array}{c} \text{general}\;\text{curvature}=\text{profile}\;\text{curvature}+\text{plan}\;\text{curvature}. \end{array}$$

*Tangential Curvature*. Tangential curvature was identified by Wilson and Gallant [29]. This is the curvature along the line orthogonal to the line of steepest gradient. As with plan curvature, this value indicates whether flowing substances will converge or diverge as they flow over a point [33, 34]. The equation for the tangential curvature is given as follows:

(19) $$\begin{array}{c} \text{tangential}\;\text{curvature}=-\frac{q^{2}r-2pqs+p^{2}t}{\left(p^{2}+q^{2}\right)\sqrt{1+p^{2}+q^{2}}}. \end{array}$$

*Longitudinal Curvature*. Identified by Wood [32, 33], this is conceptually similar to the curvature of the line of intersection between the surface and the plane defined by the slope and aspect direction. It is interpreted in the same manner as profile curvature, in that it tells whether a flowing substance will be accelerating or decelerating as it goes over a point. The following equation shows how longitude curvature is calculated.

(20) $$\begin{array}{c} \text{longitudinal}\;\text{curvature}=-2\left\{\frac{p^{2\;}r+pqs+q^{2}t}{p^{2}+q^{2}}\right\}. \end{array}$$

*Cross Section Curvature*. Cross section curvature was identified by [32, 35]; this is conceptually similar to the curvature of the line of intersection between the surface and plane defined by the slope normal and aspect direction. It is interpreted in the same way as plan curvature, in that it tells us whether a flowing substance will be converging or diverging as it goes over the point:

(21) $$\begin{array}{c} \text{cross}\;\text{section}\;\text{curvature}=2\left\{\frac{q^{2}r-pqs+tp^{2\;}}{p^{2}+q^{2}}\right\}. \end{array}$$

Hence, each type of curvature could be convex, concave, or flat. The curvature values consider the corner stone in the curvature shape estimation using (15).

*Diagonal Line Lengths*. Diagonal line is the line passing through the central cell from the two-corner neighbor cells (Figure 4). By calculating *f*_*x*_ and *f*_*y*_ through the neighboring cells and using Pythagorean theorem in calculating the hypotenuse, the diagonal length of the values of the center cell can be obtained. It is determined by the horizontal and vertical deltas, as shown in (22).  Figure 5(j) shows the length of diagonal line for Penang island:

(22) $$\begin{array}{c} \text{diagonal}\;\text{length}=\sqrt{f_{x}^{2}+f_{y}^{2}}. \end{array}$$

*Surface Area*. There are a variety of methods in the literature for measuring surface irregularity by using DEM data [36, 37]. An estimate of the surface area could also be derived from the slope and the slope aspects within a cell [38]. For this study, the Berry method was used. The surface area is equal to the planimetric area. Its value reflects the topographic surface area within that cell. There are two conditions for calculation as indicated in (23) and (24). The surface area map is shown in Figure 5(k). Consider the following:

(a) if adjustment factor value = 1,
  (23) $$\begin{array}{c} \text{surface}\;\text{area}=\;C^{2} \end{array}$$
(b) if adjustment factor value > 1,
  (24) $$\begin{array}{c} \text{surface}\;\text{area}=\frac{C^{2}}{\cos\left(\text{slope}\;\text{angle}\right)}, \end{array}$$
  where the adjustment  factor = 1/cos⁡ ⁡(slope  angle) and *c* is the cell area.

*Surface Roughness*. Surface roughness is useful as it reflects numerous geophysical parameters, such as landform characteristics, distribution of crenulations, and degree of erosivity. A number of methods have been proposed for the definition, calculation, and application of surface roughness based on the different types of parameters required for various analyses [39–41]. For this study, (25) was used to extract the surface roughness and Figure 5(l) shows the map of surface roughness:

(25) $$\begin{array}{c} \text{surface}\;\text{roughness}=\sqrt{\frac{1}{N}\sum_{i}^{N}{\left(S_{i}-\overset{-}{S}\right)}^{2}}. \end{array}$$

*Rugosity*. This factor is the ratio of the surface area to the planar area across the neighbourhood of the central pixel which is *Z*_5_ (Figure 4). Using this method, flat areas will have a rugosity value near to 1, while high relief areas will exhibit higher values of rugosity [42], as shown in (26).  Figure 5(m) shows the map of rugosity of Penang island. Cosider

(26) rugosity =  surface  area  of  3  ×  3  neighborhood  windowsplane  area  of  3  ×  3  neighborhood  windows.

### 4.2. Determination of Important Factors

Two methods are implemented to determine the important factors for landslide analysis. They are weight determination using Zhou method and classification accuracy method.

#### 4.2.1. Weight Determination and Factor Rating Using Zhou Method

As stated previously, the back-propagating approach is suitable to be used for landslide application in order to determine the weight of each input factor. Zhou (1999) described a method for weight determination using back-propagation. The same method is adopted in this study. The effect of an output *O*_*j*_ from the hidden layer nodes  *j* on the output *O*_*k*_ from an output layer node  *k* can be represented by the partial derivative of *O*_*k*_ with respect to *O*_*j*_ shown as follows:

(27) $$\begin{array}{c} \frac{do_{k}}{do_{j}}=\tilde{f}\left({\text{net}}_{k}\right)\frac{d\left({\text{net}}_{k}\right)}{do_{j}}=\;\tilde{f}\left({\text{net}}_{k}\right)w_{jk}. \end{array}$$

Equation (27) can have both negative and positive values. Weight importance of node  *j*  relative to another node  *j*_*o*_  in the hidden layer may be calculated as the ratio of the absolute values derived from the following eqution:

(28) $$\begin{array}{c} \frac{\left|do_{k}/do_{j}\right|}{\left|do_{k}/do_{jo}\right|}=\left|\frac{\tilde{f}\left({\text{net}}_{k}\right)w_{jk}}{\tilde{f}\left({\text{net}}_{k}\right)w_{jok}}\right|=\;\left|\frac{w_{jk}}{w_{jok}}\right|, \end{array}$$

where *w*_*jok*_ is another weight in *w*_*jk*_ other than *w*_*ik*_. Equation (28) shows that with respect to a particular node *k* in the output layer, the relative importance of node *j* in the hidden layer is proportional to the absolute value of the weight on its connection to the node *k* in the output layer. Eqution (28) can be used to compute the importance of the node in the output layer when it has one output. For the neural network with more than one output, the following equation is used:

(29) $$\begin{array}{c} w_{jok}=\frac{1}{J}\cdot \sum_{j=1}^{J}\left|w_{jk}\right|. \end{array}$$

The normalized importance of the node *j* in the hidden layer with respect to node *k* in the output layer is given as follows:

(30) $$\begin{array}{c} t_{jk}=\frac{j\left|w_{jk}\right|}{\sum_{j=1}^{J}\left|w_{jk}\right|}. \end{array}$$

The total importance of the all nodes in the hidden layer with respect to the same node is given by the following equation:

(31) $$\begin{array}{c} \sum_{j=1}^{J}t_{jk}=J. \end{array}$$

The importance of each node in the hidden layer with respect to all of the nodes in the output layer can be calculated as given by the following equation:

(32) $$\begin{array}{c} t_{j}=\frac{1}{K}\sum_{j=1}^{K}t_{jk}. \end{array}$$

Similar to (29), with respect to node *j* in the hidden layer, the normalized importance of the node *i* in the input layer can be defined as follows:

(33) $$\begin{array}{c} s_{ij}=\frac{I\cdot \left|\omega_{ij}\right|}{\sum_{i=1}^{I}\omega_{ij}}. \end{array}$$

With respect to the hidden layer, the overall importance of node *i* is given by the following equation:

(34) $$\begin{array}{c} S_{i}=\frac{1}{J}\cdot \sum_{j=1}^{J}S_{ij}. \end{array}$$

Correspondingly, the overall importance of the input node *i* with respect to the output node *k* is given by the following equation:

(35) $$\begin{array}{c} {St}_{i}=\frac{1}{J}\cdot \sum_{j=1}^{J}S_{ij}\cdot t_{j}. \end{array}$$

**Figure 6:**
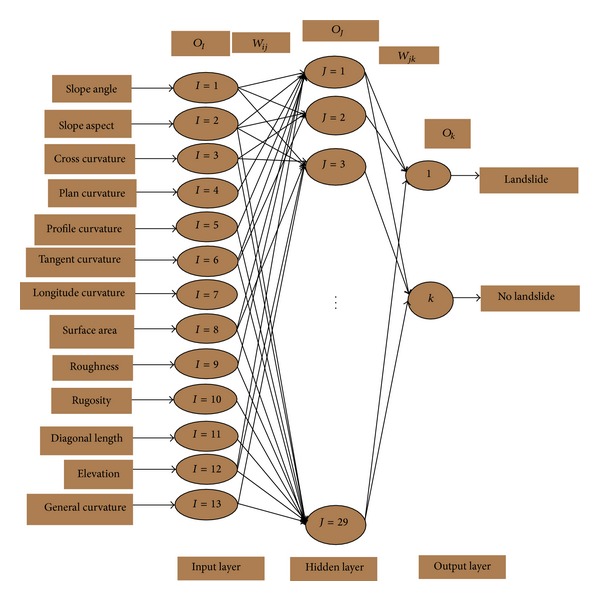
General structure of MLP model.

For this study, the structure of the MLP with the back- propagation algorithm chosen as the training algorithm (Figure 6) was selected to be 13 × 29 × 2. This neural network consists of three layers, where the first is the input layer, the second is the hidden layer, and the third is the output layer. Each neuron of input layer represents one input factor connected with the input layer. The number of the hidden layer neurons chosen is 29 in this research work. In addition, the output layer has two neurons representing landslides and no landslides.

The back-propagation algorithm connects the three layers of the MLP to minimize the error between the predicted output and the actual output as in (5). This algorithm, learning rate, momentum, and epoch number control the performance of the neural network. The momentum, learning rate, and performance rate were set to 0.9, 0.01, and 0.1, respectively. In addition, the epoch number was set to 1000. If the neural network performance could not reach the mean square error (MSE) of 0.1, the network will stop after 1000 epochs. Therefore, no overfitting occurs during the training.

Thirteen topographic factors were entered into the neural network at the same time. The average of the weights of each factor after 10 times of training for the same data sets was taken. The weight normalization which tells the importance of each factor was done by calculating the mean weight value of each factor after 10 cycles of training and then dividing the values by the minimum mean weight value among the thirteen factors. The factors with highest rating are taken as factors with highest importance.

#### 4.2.2. Factors Rating Using Classification Accuracy

Rating of the importance factors by using the neural network classification accuracy is being applied for the first time on landslides hazard analysis. It is carried out in the second stage of the training by entering one of the thirteen factors to the neural network at one time and checking the classification accuracy of each factor. The process repeats for each of the thirteen factors.

The accuracy of the neural network after each time of training was calculated by using the following equation:

(36) $$\begin{array}{c} \text{accuracy}=\frac{\text{number}\;\text{of}\;\text{samples}\;\text{correctly}\;\text{classified}\;}{\text{total}\;\text{number}\;\text{of}\;\text{samples}}. \end{array}$$

This step was repeated 10 times for every single factor and data set. After that, the accuracy of the neural network for every factor was calculated by taking the average of the highest and lowest accuracy from the 10 sets of training data. The accuracy of the neural network for every factor after training has been considered for factor rate normalization. In addition to that, each factor having 70% accuracy and more is considered as a good factor and will be used on the second stage of classification. Otherwise, the factor is considered as not good enough and therefore is ignored. These ignored factors actually affect the overall neural network performance by reducing the accuracy of classification.

## 5. Data Preparation and Classification Performance

An effective intelligent system requires a comprehensive data set. Therefore, 137570 pixel data were selected from each factor in this analysis, where 68786 pixels represent landslides and 68786 pixels represent no landslides. The data were normalized to range between 0 and 1 for each of the factors individually based on the following equation:

(37) normalised  pixel(i) =pixel(i)−minimum  pixel(I)maximum  pixel(I)−minimum  pixel(I),

where the pixel(*i*) is the pixel to be normalized and (*I*) is the minimum or the maximum pixels value for every single factor. The intelligent system target (landslides history) is represented by 1 for landslides and 0 for no landslides.

For the intelligence performance, this study employs a 10-fold cross validation method to arrange the number of the data for training and testing sets [43]. In this method, the data are partitioned into 10 sized segments or folds. Each fold consists of 13757 pixels, which are divided by half to landslides and no landslides. Ten iterations of training and testing are performed. In each iteration, one part of the data is held out for testing while the remaining 9 parts are used for training. The MLP layers weight and output accuracy were noted.

## 6. Results and Discussion

Thirteen factors were involved in this study. They were extracted from the Penang DEM map using (7) to (26). Topographic factor maps were then verified by comparing the extracted maps using different software such as ArcView, IDRISI, and MapInfo. Satisfactory results were achieved.  Table 1 shows the weights produced based on Zhou method whereas weights for all topographic factors obtained using classification accuracy are presented in Table 2. Training the data for 10 times using Zhou's method has produced almost similar values of the weight after each training with small differences on values as observed in Table 1. In addition, the standard deviations distribute from 0.006 to 0.027. In the final analysis, the seven factors with high rating are the diagonal length, longitude curvature, cross section curvature, general curvature, surface area, slope angle, and slope aspect. Out of the 13 factors, tangential curvature has a minimum value of 1.0 and the slope angle has a maximum value of 1.42.

The accuracy classification method result (Table 2) gives the details on the accuracy of every single factor for 10 different data sets. The standard deviation distribution shows low values 0.0341 to 0.1829. The ten data sets have produced the weights with small changes among them.

There are no large changes on the factor's importance (weights) on the training data. The factor's importance was determined through the training stage. Diagonal length and tangential curvature were the most and least important factors, respectively, during the training and testing stage.

It is observed that there were 8 factors having more than 70% of classification accuracy. Coincidentally, they are the same factors with the highest rate using Zhou's method. Therefore, they were chosen for further neural network training and testing. At this stage, a combination of the good factors was employed to improve the performance of the neural network. The number of inputs for the neural network was equivalent to the number of important factors while the number of outputs was two, which represents the landslide prone and not landslide prone.

Table 3 depicts the classification performance for all of the factors and the eight important factors. The results have shown improvement in the classification where the average classification is 82.00% using all 13 factors and improve to 85.3% when only 8 important factors are used. The factors with low rates are roughness, plan curvature, tangent curvature, cross curvature and general curvature, and they have negative effect on the classification accuracy and can therefore be ignored.

## 7. Conclusion

The classification of the landslide can be improved by choosing the suitable factors. Zhou method and classification accuracy method are proven to be efficient in the selection of important factor in this study area. By ignoring the 5 unimportant factors, that is, roughness, plan curvature, tangent curvature, cross curvature and general curvature, the performance of classification has improved and the complexity of the network has been decreased. Both methods have shown a consistent agreement on the eight significant and important factors. These are profile curvature, rugosity, slope aspect, elevation, slope angle, diagonal length, longitude curvature, and surface area. In descending order, the ratings of the important factors for Zhou's method were slope angle, slope aspect, profile curvature, diagonal length, elevation, surface area, rugosity, and longitude curvature. The least significant factor is roughness. In the classification accuracy method, the slope angle has the highest rate and the roughness has the lowest rate. Meanwhile, the rest of the factors were rated in between these two.

## Figures and Tables

**Table 1:** Weights based on Zhou's method.

	Number of test										SD	Mean	Weight
	1	2	3	4	5	6	7	8	9	10			
Diagonal length	0.1876	0.1616	0.1296	0.1376	0.1686	0.1446	0.1626	0.1746	0.1476	0.1376	0.0187	0.1552	1.1669
Slope angle	0.2002	0.1822	0.2012	0.1792	0.1802	0.1682	0.1812	0.2092	0.2002	0.1822	0.0132	0.1884	**1.4165**
Surface area	0.1369	0.1509	0.1429	0.1219	0.1509	0.1569	0.1449	0.1429	0.1339	0.1759	0.0145	0.1458	1.0962
Cross curvature	0.1279	0.1609	0.1249	0.1549	0.1409	0.1459	0.1229	0.1579	0.1359	0.1259	0.0146	0.1398	1.0511
Plan curvature	0.1478	0.1308	0.1198	0.1298	0.1458	0.1438	0.1188	0.1448	0.1348	0.1198	0.0116	0.1336	1.0045
Tangent curvature	0.1497	0.1327	0.1217	0.1317	0.1477	0.1457	0.1207	0.1467	0.1367	0.1217	0.0115	0.1355	1.0187
Longitude curvature	0.1523	0.1573	0.1513	0.1463	0.1493	0.1323	0.1303	0.1533	0.1463	0.1273	0.0107	0.1446	1.0872
Roughness	0.1485	0.1435	0.1155	0.1295	0.1375	0.1335	0.1145	0.1335	0.1465	0.1275	0.0118	0.1330	**1.0000**
Slope aspect	0.1790	0.1690	0.1670	0.1640	0.1640	0.2450	0.2110	0.1630	0.1740	0.1840	0.0264	0.1820	1.3684
Rugosity	0.1436	0.1546	0.1416	0.1386	0.1446	0.1436	0.1466	0.1576	0.1396	0.1416	0.0062	0.1452	1.0917
General curvature	0.1554	0.1514	0.1444	0.1444	0.1274	0.1364	0.1234	0.1354	0.1374	0.1324	0.0101	0.1388	1.0436
Elevation	0.1494	0.1624	0.1274	0.1454	0.1444	0.1494	0.1354	0.1564	0.1444	0.1534	0.0101	0.1468	1.1037
Profile curvature	0.1769	0.1679	0.1849	0.1789	0.1539	0.1789	0.1469	0.1549	0.1899	0.1449	0.0164	0.1678	1.2616

The bold values represents the minimum and the maximum values (factors).

**Table 2:** Weight based on classification accuracy method.

	Number of test										SD	Mean	Weight
	1	2	3	4	5	6	7	8	9	10			
Slope aspect	76.69	76.64	76.34	76.72	76.73	76.40	76.68	76.29	76.68	76.34	0.1829	76.55	1.261
Surface area	74.76	74.74	74.72	74.77	74.86	74.77	74.75	74.71	74.71	74.70	0.0458	74.75	1.231
Slope angle	77.48	77.43	77.48	77.47	77.64	77.51	77.31	77.41	77.54	77.33	0.0980	77.46	**1.280**
Tangent curvature	61.04	61.05	61.11	61.00	61.04	61.02	61.00	61.04	61.07	61.01	0.0341	61.04	1.009
Rugosity	72.50	72.48	72.51	72.50	72.56	72.50	72.56	72.45	72.54	72.54	0.0342	72.51	1.191
Profile curvature	70.87	70.79	70.93	70.80	70.89	70.81	70.88	70.75	70.84	70.83	0.0534	70.84	1.171
Longitude curvature	76.32	76.29	76.30	76.31	76.38	76.30	76.35	76.26	76.32	76.25	0.0385	76.31	1.262
Roughness	60.44	60.44	60.46	60.41	60.53	60.42	60.57	60.43	60.41	60.45	0.0532	60.46	**1.000**
Diagonal length	72.20	72.15	72.19	72.23	72.24	72.16	72.26	72.13	72.24	72.16	0.0441	72.20	1.194
Cross curvature	67.97	67.97	68.01	68.03	68.04	67.99	67.99	67.94	67.96	67.92	0.0366	67.98	1.122
Plan curvature	60.67	60.60	60.63	60.59	60.70	60.67	60.68	60.65	60.65	60.65	0.0343	60.65	1.003
Elevation	72.88	72.86	72.88	72.92	72.97	72.93	72.92	72.80	72.89	72.86	0.0472	72.89	1.201
General curvature	69.60	69.53	69.67	69.59	69.65	69.59	69.63	69.58	69.62	69.62	0.0387	69.61	1.153

The bold values represents the minimum and the maximum values (factors).

**Table 3:** Testing and training accuracy in % for both all and important factors.

Classification method	Accuracy (Before factors selection, all 13 factors)	Accuracy (After factors selection, 8 important factors)
MLP with LM	82.00%	85.3%
